# Beyond Good and Bad: Rethinking Solidarity and Coercion in Public Health

**DOI:** 10.1080/15265161.2026.2632018

**Published:** 2026-02-27

**Authors:** Tess Johnson, Safura Abdool Karim, Diego S. Silva

**Affiliations:** 1 Ethox Centre, Oxford Population Health, University of Oxford, UK; 2 Pandemic Sciences Institute, University of Oxford, UK; 3 Berman Institute of Bioethics, Johns Hopkins University, USA; 4 Center for the AIDS Programme of Research in South Africa, University of Kwazulu-Natal, South Africa; 5 Sydney Health Ethics, School of Public Health, University of Sydney, Australia

**Keywords:** Public health ethics, coercion, solidarity, infectious disease, public health emergencies

## Abstract

We often use certain terms as if, in using them, they contain a decided moral judgement of an action. Especially in public health ethics, this is not always the case, as shown most starkly by recent (mis) use of the terms ‘solidarity’ and ‘coercion’ to label, and thereby judge, public health actions responding to the COVID-19 pandemic. We analyse the terms solidarity and coercion, and argue that they cannot be used alone as moral judgements of public health actions. Rather, they are better considered as descriptive terms that are merely frequent proxies for normative terms such as justice or utility. We illustrate our argument by reference to three case studies: school reopenings in the USA, mandatory isolation measures in the UK, and vaccine distribution within the EU.

## Introduction

Public health policy makers, practitioners and researchers – including ethicists – use certain terms as if the moral valence of said terms is set, well-understood, or shared by various actors. For example, if we ask the reader whether equity or trust are, all things considered, good or bad ‘things’, at first blush they’ll likely respond ‘good!’ If we ask the reader whether autonomy is, all things considered, a good or bad ‘thing’ (and they’ve read enough of the public health ethics literature over the last 20 years), they’ll likely remark ‘good’ but less enthusiastically than before. Some terms, like paternalism, might elicit a ‘it depends’ or ‘bad-ish’ answer to the same line of questioning. This (very) simple thought experiment isn’t meant as a conclusion in favour of emotivism but merely suggests that our claim at the outset, that terms are often used as if their moral valence is set, well-understood, or shared, is *prima facie* plausible.

One problem with this phenomenon is that it obfuscates the relationship between the potential normativity of ideas and their descriptive function. For example, a description of an instance of an autonomous action (e.g., providing informed consent to participate in a study) is confused with moral approbation of autonomy (e.g., informed consent is good). This conflation between the use of terms to describe action and the moral valence or normativity of those terms can lead to a deeper problem: the use of terms with their purported valence set stands in for argumentation in favour of or against a reason for action, or in judgement of past acts. In areas like public health ethics, this problem could lead to inadequately defended policy choices, and possibly, ethically inappropriate public health policy as a result. If this problem exists, there are at least two possible paths to take: first, one can reject the idea that some terms can ever have any moral valence at all; this is a strong position which we set aside here. Rather, we’ll take the second path, arguing that in public health ethics in particular it is important to understand how terms are appropriately used descriptively, and why we are better off using more obviously morally-valent terms to come to a reasoned ethical judgement of a public health action.

Public health responses to infectious disease outbreaks, as we’ve exited the pandemic phase of existing with COVID-19, provide us with important case studies that allow us to analyse how: (a) moral terms are often used as if their valence is set; and (b) the invocation of such terms stands in for moral reasoning and evaluation. Two terms in particular are worth noting with regard to how their meanings and usage as justifications or condemnations for public health actions fluctuated throughout the COVID-19 pandemic: solidarity and coercion. We have chosen these terms, as they were outliers in how much they were used during the COVID-19 pandemic, and how much work they did in acting as justifications for particularly controversial actions, including lockdowns, vaccine procurement deals, and changes in education. Herein we make the modest argument that the moral permissibility of public health measures does not hinge on the purported rightness of solidarity and wrongness of coercion, but rather on more fundamental and obviously normative questions about effectiveness of the measure at promoting a primary good (health), the measure’s justice implications, etc. In other words, we argue that the labels ‘solidaristic’ and ‘coercive’ in themselves do not determine the moral status of an action. We will not wade into the ongoing debate about the usage of these terms in purely theoretical, normative ethics, but rather defend our core argument only within the scope of applied public health ethics specifically. In the context of public health, we acknowledge that it is often the case that solidaristic actions are also just and effective at promoting health (and therefore good). Indeed, recent work points out how ‘parasitical solidarity’ fails to support the oppressed or fight injustice (Weiss 2025). Similarly, it is often the case that actions that are coercive are also unjust and ineffective at promoting health (and therefore bad). Yet, the propensity for solidarity to correlate to ‘good’ and coercion to ‘bad’ does not establish that this is always the case; treating these ideas as if their moral valence is established from the outset obfuscates the moral debates we should be having in public health.

In the first section of the paper, we present conceptual overviews of solidarity and coercion and suggest that they can be used as descriptors of phenomena and that their moral valence is less established than it might seem at first blush. Afterward, we support this claim by outlining and then analysing three cases of solidaristic and coercive public health actions from the COVID-19 pandemic. We suggest that the measures described as solidaristic or coercive require further analysis to determine whether the actions were morally permissible or not. Other moral concepts or terms used in ethical analysis such as justice may be intrinsically reason-giving in a way that solidarity and coercion are not. Whilst we do not have the space here to characterise the distinction between normative and non-normative terms as a whole, we do make brief reference to metaethical theory on non-naturalism, and we hope that this work paves the way for further research in the area and helps ethicists assess the appropriateness of normative terms used in ethical analysis.

### 1 The Valences of Solidarity and Coercion

During the COVID-19 pandemic, the concepts of solidarity and coercion came up frequently in political and public discussion—they were heavily used as pieces of rhetoric. Phrases like “we’re all in this together” were used to justify not only self-isolation orders, but other measures involving sacrifices to protect others, such as mask mandates, lockdowns and travel bans (UK Home Office 2020; Silva et al. 2021). Publics around the world were encouraged to stay at home in order to “stop the spread” (Massachusetts Department of Public Health n.d.). Some were required to show apps with proof of vaccination status to enter restaurants, gyms, or clubs, tackling COVID-19 as a “shared threat” (Department of Health and Social Care 2020; Mills 2021). Sometimes, these labels were manipulated or applied where the concept did not hold—that is, an action was called ‘solidaristic’ merely as part of political rhetoric, without it actually having that characteristic. However, as we will discuss further in Section 2 of this paper, there were cases where the labels or signifiers ‘coercive’ and ‘solidaristic’ were used both as rhetoric and in a descriptively appropriate way–where there was indeed a signified (actual coercion or solidarity) that they referred to. In the cases we will examine, the signified solidarity did not render the action (more) right, and the signified coercion did not render the action (more) wrong.

Generally, during the pandemic, solidarity was framed as positive, and coercion as negative. Solidaristic acts, like countries sharing epidemiological information on the outbreak of new strains, were regarded as morally praiseworthy, or even morally required. Typically, the bioethics and philosophical literature also attaches positive normative valence to solidarity (Prainsack and Buyx 2012; Illingworth and Parmet 2012; Jennings 2017) and negative normative valence to coercion (Ryan 1980; Wertheimer 1987). As a result, we may often consider ‘solidaristic’ practices as good, and ‘coercive’ practices as bad, without further reflection beyond that labelling. This may be the case even where there are multiple different conceptions or definitions of the terms at play, as is the case within public health ethics. Similarly, solidarity may be used as a justification or positive mitigating factor for a practice otherwise thought bad; coercion may be considered to undermine or contribute *pro tanto* wrongness to a practice otherwise thought good.

Arguments for the distinction between description and normative valence are, of course, quite old in metaethics—we might refer to Moore’s non-naturalism in the *Principia Ethica*, for instance (1903 [1993]). In bioethics, however, the debate is live. As Mertz and colleagues highlight, it is an essential puzzle in the conduct of empirical bioethics research, at the heart of the question about how what policymakers or the public describe as right or wrong in an interview, say, and what is in fact right or wrong (Mertz et al. 2023). Perception of rightness and the existence of rightness can come apart. In a parallel vein, the existence of a characteristic of an action, say, solidarity, and the rightness of that action can come apart. Emerging work on ‘inclusive’ and ‘exclusive’ solidarity has the capacity to highlight this disconnect (Prainsack 2026), as does emerging work on ‘parasitic’ and ‘misappropriated’ solidarity (Weiss 2025).

In the proceeding two subsections, we outline the potential normative ambiguity of both terms.

#### 4.3 Solidarity

We offer a preliminary definition of solidaristic action here as action that identifies a group with a shared characteristic, interest, or consequence, and willingly takes on costs to promote their shared best interests. This definition is not identical to, but aligns with, that proposed by other influential work in the area that emphasised group membership, shared status, and interest promotion (of the vulnerable), without being a normative definition (Buyx and Prainsack 2017; Prainsack 2026). The ideas behind this definition can be traced back in the history of the term. Solidarity is often imbued with different meanings depending on time, culture, and context. This may be, in part, attributable to the way that authorities may reactively reach for solidarity in times of crisis or difficulty. This turn to solidarity in challenging times can be seen with the increased references to solidarity during COVID-19, or even in early discussions about climate change or refugee crises, where greater solidarity is indeed demanded by circumstance (Buyx and Prainsack 2017; Illingworth and Parmet 2017). The breadth of solidarity’s apparent relevance across public health and other areas may also be an artefact of the lack of a clear definition, and the vagueness that some have criticised as emptiness (Buyx and Prainsack 2017). Consequently, solidarity may often be descriptive of practices and actions rather than afforded an intrinsic meaning.

The evolution of solidarity as a broad pro-social umbrella that encompasses ideas such as togetherness, unity and community may be seen as a result of its historical uses in responses to different political, social and religious movements. The concept of solidarity has its roots in the Roman Law concepts of obligations *in solidum* that were contractual in nature but constructed as a liability “all together” (Sangiovanni 2015). Solidarity’s social character developed in reaction to the deterioration of communities–serving as a basis and foundation for counteracting that deterioration through social unity. As a political concept, solidarity has its origins in the French Revolution’s term “*solidarité*”. Becoming a concept embroiled with ideas of class, the notion of “togetherness” arises in opposition to exploitation as well as in collective action, in the form of revolution, as a manifestation of the response to these inequities and exploitation. Later, there is greater emphasis on joint social production (Sangiovanni 2015). Durkheim describes the change in the nature of solidarity as being from a “mechanical solidarity” anchored in “sameness” to an “organic solidarity” emerging in workers’ movements based on recognition of the dependency people have on others (1997). In contrast, the Christian notions of solidarity–particularly those rooted in Catholicism and Aquilian thought–focus less on a shared sense of communality, recognising the interconnectedness of and shared similarity as being a result of Christian values (Prainsack and Buyx 2016; Buyx and Prainsack 2017). These notions of solidarity have developed links to social justice.

In the 21^st^ century, solidarity has come to play a very different role, particularly within the context of public and global health. Specifically in the bioethics literature, solidarity has been drawn on in a different way–primarily to justify intervention or actions for the improvement of collective or population-scale health (Prainsack and Buyx 2012). In the context of global health, solidarity is frequently used to provide a normative basis to demand that wealthier countries provide assistance to low- and middle-income countries to equalise health disparities. Solidarity is sometimes invoked to justify resource allocation and rationing decisions in healthcare systems that are aimed to achieve justice and equity. In the latter two instances, solidarity is specifically linked to the vulnerability of the persons or groups affected by the decisions by Prainsack and Buyx 2016. It is linked to vulnerability of immigrants in relation to accessing health services, by Illingworth and Parmet 2017. Each of these interpretations thus relies on a specific understanding of what justice requires in relation to the pursuit of equality or addressing the needs of the worst off or most vulnerable as a priority. In each of these contexts, solidarity plays a slightly different role and takes on a different character. In public health and health care systems, solidarity can act mechanistically as a means to justify certain actions. Bioethicists have invoked solidarity to justify the use of, for example, vaccine mandates on the basis that such mandates may rightly impose burdens on individuals to assist the more vulnerable members of their community in protecting their health (West-Oram 2021; Ewuoso et al. 2022; Yeh 2022).

In the global health context, solidarity is imbued in both the academic literature and in political rhetoric with a positive moral valence that forms the basis for obligations and demands on wealthier countries to engage in cooperative action that supports and assists poorer countries for the promotion and protection of global health where threatened (West-Oram and Buyx 2017; de Campos-Rudinsky 2023; West-Oram 2021). In this sense, solidarity is sometimes used in ethical analysis as a standalone label to justify or compel action for the betterment of public health. As a review conducted by Buyx and Prainsack (2017, 9) outlines, this may build on a fear in global health settings that “solidarity itself was assumed to be in danger of disappearing.” Correspondingly, critiques of the use of solidarity as a concept focus more on whether the requirements of solidarity are really fulfilled through a particular action rather than whether they necessarily *should* be fulfilled—i.e., whether an action labelled as solidaristic is by virtue of its solidarity, a good thing. The assumption of solidarity’s positive moral valence influences its adoption, too, in policy decision-making (Prainsack and Buyx 2016; Rahman-Shepherd et al. 2021), along with its use in global health instruments and governance frameworks (de Campos-Rudinsky 2023; WHO Pandemic Agreement 2025). Where there is no further ethical analysis of a proposed solidaristic action, the reference to solidarity in policy particularly may mask an underlying failure to ethically assess actions that include some and exclude others (Sangiovanni 2007; Prainsack and Buyx 2012; Illingworth and Parmet 2015). As Prainsack notes (2026), exclusive solidarity, which is confined to small groups, often compromises inclusive solidarity, which extends to wider populations. Illingworth and Parmet (2015) note a similar caution, recognising the potential of solidarity to “foster exclusionary tendencies” (p. 65). An action that promotes the wellbeing of a small, privileged group is (inclusively) solidaristic; an action that promotes the wellbeing of the globe is (exclusively) solidaristic. Yet, their moral status might not be equivalent. If so, then the moral status of the action is not determined by its solidarity, and solidarity is not always to be promoted; solidarity does not necessarily have positive moral valence.

In the context of COVID-19, solidarity has become synonymous with communities and states working together, and the failure to do so, framed as a failure to act in solidarity (Greer 2020; Rahman-Shepherd et al. 2021; Kapadia 2022). That failure is almost universally viewed negatively and is also linked to the persistence and worsening of inequities and global injustice. The expectation was that solidarity required states to politically and financially prioritise interventions against COVID-19 that served the world’s population. Director-General of the World Health Organisation, Dr Tedros Ghebreyesus repeatedly appealed to solidarity as the normative basis for the pandemic response, in one instance linking solidarity (and an array of specific obligations emanating therefrom) to the success of controlling COVID-19, “[W]e cannot defeat this outbreak without solidarity – political solidarity, technical solidarity and financial solidarity.” (Rahman-Shepherd et al. 2021, n.p.) We might ask whether the move in these and other such discussions from an initial focus on global health justice to, instead, solidarity, was appropriate, or whether it instead merely allows the catchier language of solidarity to mask the underlying moral question of whether an action promotes justice or not.

Amidst these repeated appeals to solidarity are practices of solidarity that may be observed which are less helpful to global health but may very well promote a state’s or a region’s own particular public health objectives—cases of exclusive or group-centric solidarity. For example, there were efforts to promote solidarity in the response of the European Union (EU) wherein the EU Commission re-interpreted provisions related to export bans to permit them for medical supplies and COVID-19 countermeasures, provided that the bans did not cause or aggravate shortages of essential goods in the EU (Greer 2020). This act of solidarity aimed to protect access to these goods within the membership of the EU but gave no consideration to those outside of the EU, as we will explore more fully in the section below. A similar kind of separation between who is owed solidarity and who is not can be observed in the Pandemic Agreement negotiations (Hosseini 2023; WHO Pandemic Agreement 2025): the Pandemic Agreement is positioned as a fundamentally solidaristic instrument, yet solidarity has been used by different groups to demand different things. Wealthier countries have appealed to solidarity to demand more expansive information and pathogen sharing provisions while fighting against the inclusion of measures which African states argue are anchored in solidarity such as stronger duties of mutual assistance and benefit sharing (Silva and Smith 2022).

These examples gesture at another underlying problem in moralising solidarity as being positive–that solidarity hinges on a sense of sameness and commonality which may easily be instrumentalised to exclude those who are not “part of” or not sufficiently the “same” – this is the risk of exacerbating exclusionary tendencies that Illingworth & Parmet (2015) allude to. This harkens back to the mechanistic solidarity Durkheim described as pre-dating the organic solidarity of workers’ movements (1997). However, even within the history of solidarity, it being raised in opposition to has necessarily resulted in a division between those whom we act in solidarity “with” and those we act in solidarity “against”. In the same manner that exploited workers could be acting in solidarity with each other, the bourgeois class who enacted the exploitation acted in solidarity with each other. This raises questions as to whether solidarity should be considered to have positive moral valence.

#### 1.2 Coercion

The term ‘coercion’ is commonly used to label actions where someone (a coercer) has made someone else (a 8omewh) do something, usually through force, threat, or exploitation of a power differential. This is the preliminary definition of coercion that we offer here, too. In contrast to much of the ethics literature, where coercion is moralised as wrong by definition, ours is intended to merely denote a mechanism of action. In the public health context, during COVID-19, the label has been applied, for instance, to interventions including mask mandates or stay-at-home orders (Massachusetts Department of Public Health n.d.), public health ‘police power’ used by states to enforce social distancing (Mills 2020), and ‘vaccine passports’ (Somerville 2021). These measures were often framed as necessary evils with the end-goal of protecting those at risk of more severe health outcomes from infection justifying the measure, overall. We would not go so far as to call them evils to begin with.

For the most part, common usage of the term coercion through the COVID-19 pandemic aligned with recent scholarship on it in the spaces of political philosophy and public health ethics. Coercion that doesn’t use physical force is often defined in the literature as the use of pressure on a 8omewh’s will to make them act in accordance with the coercer’s will (Nozick 1969; Wertheimer 1987). Less often, it is defined as the leveraging of power differentials between coercer and 8omewh to reduce the 8omewh’s practical possibilities for action (Anderson 2010). The former definition might apply for example when an armed robber threatens a victim with violence if they don’t give up their wallet. The latter definition might be more apt when talking about the direct removal of an option for action, say when the state imprisons someone who has broken criminal law.^1^

There has been debate about whether coercion should be moralised, whether it has normative valence. There are two aspects to such moralisation or normative valence questions. Alan Wertheimer, a key coercion scholar, is concerned with both aspects: first, whether in order to apply a label of coercion, we must make complex moral judgements (that is, whether ascribing coercion is based on both moral and empirical premises), and second, whether coercion always has implications for our moral judgements.^2^ Scholars who focus only on the moral implications of coercion and not on whether moral judgements are required earlier on for ascribing the label might be thought to have a partially normative or moralised account. They ask merely whether, once it is ascribed, the label of coercion is necessary and sufficient to render an action wrong. For those who hold this is necessary and sufficient, coercion is absolutely wrong. For those who hold the ascription of coercion to an action is not enough to always render it wrong, coerciveness may be *pro tanto* wrong, but the coercer’s action could be right, all things considered, on the basis of other contributing moral reasons in favour of the action (Ryan 1980). Most common views of coercion in the public health ethics literature hold it to be *pro tanto* wrong, having a negative moral valence that can be outweighed by other considerations (Giubilini 2023). We disagree with this view, arguing instead, that the term is itself morally neutral, which is a stronger position than taken in the public health ethics literature on coercion to date.

For those who see coercion as morally neutral, it may be neutral either merely in the sense of its not having moral implications for a coercive action, or also regarding there not being prior moral judgement of an action required before ascribing the label of coercion to an action to begin with. The latter sense is a fully descriptive rather than normative account of coercion. For these scholars, a coercive action may be either right or wrong depending entirely on other factors. Those who challenge a presumption in favour of liberty (and thus any wrongness of coercion as a result of liberty infringement (Zimmerman 2002)) fall within this group, as do theorists who see coercion as a mechanistic description of a relationship between coercer and 9omewh, which may be right or wrong depending on other factors (Anderson 2010). Such ‘mechanistic’ descriptions may be particularly useful for characterising relationships between groups with easily defined and legitimate power differentials, such as a state and its citizens/residents. They follow a long tradition of ethical analysis of state action that does not rely on the mere ascription of a label of coercion for deriving moral content. Hobbes is famous for his argument that to avoid a state of chaos, a social contract is needed where naturally war-like humans give up our right to self-governance, acquiescing instead to be ruled by a strong state in exchange for safety and order (1998 [1651]). On this view, the state need not obey its own laws, but must enforce laws equally over citizens. Coercion, then, is an important function of a state, and in some applications, coercive action protects the people from lawlessness. The state has a monopoly on this coercive power. Without fear of state power, citizens would have no reason to abide by their contracts with others. Similarly, in his *Doctrine of Right* (1996 [1797]), Kant introduces the *rechtsstaat* or juridical state which enacts punishment on those citizens who lack the independent motivation to follow the law. The function of coercion is, again, necessary to guarantee equal freedoms for citizens and prevent rights violations. On some interpretations, Kant’s view of coercion is that it is *pro tanto* wrong, because it limits freedom. Alternatively, one might rather emphasise his view that the state has a right to use coercion, derived from the duty of equal defense of citizens’ rights, which leads to a more normatively neutral understanding.

Here, we take the side of descriptivism in the conceptual debate on coercion,10omewhat on Hobbesian and Kantian views of coercion as necessary for state function. At least in the context of a state or authority setting public health policy, we see any normative valence or moralisation of a coercive action as arising from some co-occurring, other feature of the action that is actually the locus of normative valence, such as whether it produces good or bad outcomes, or whether it promotes or undermines effective public health promotion or justice. This approach avoids misclassifications of cases that normative conceptions of coercion are prone to make. For example, a descriptive account of coercion can acknowledge cases of both bad coercive acts (like the armed robber) and good coercive acts (like the enforcement of just laws or the use of force or threat in self-defense). In the armed robber case, a fully or partially moralised account might tend to apply the label of coercion according to a judgement that armed robbery is wrong; yet those applying such an account might also appeal to the coerciveness of the action in judging it to be wrong. Such circularity can be better avoided with non-normative accounts. These accounts also run into problems where it is not obvious that a coercive action is wrong on the basis of its being coercive. In the enforcement of just laws case, it seems intuitive that such an action is coercive, without reference to a prior moral judgement. It also appears not to be wrong that a state should enforce just laws, despite this coerciveness.

At this point, an objector may note that at least in the vast majority of cases, coercion seems to be wrong, and that there may instead merely be mitigating factors that render forceful self-defense and just law enforcement right (overall), despite employing (wrong) coercion. We hold that this is merely a frequent correlation of coercion and wrongness: normative valence is attached to other factors that often co-occur with coercion, such as unfair or illegitimate power differentials. In many cases (such as coercion between citizens or other moral equals) a power differential may be wrong. According to Anderson, this does not mean that coercion is always wrong, but merely that it is wrong in the context of these particular relationships (2010). By contrast, the power differential between state and citizen often seems perfectly legitimate (perhaps by virtue of the social contract, as both Hobbes and Kant argue), and as such, state-sanctioned coercion is often among the cases of good coercion (though note that the overall judgement of coercive action will still depend on other factors such as whether it fulfils the state’s obligation to protect citizens’ equal rights, etc.). This neutral approach to coercive action aligns with judgements of multiple moral theories, including, broadly speaking, consequentialism, which might judge coercive public health actions like police-enforced social distancing during the COVID-19 pandemic to be good insofar as they protect health at little cost to individuals’ overall wellbeing. It also aligns with judgements from, broadly speaking, deontological theories, which might judge well-intentioned coercion to be right, for instance in the case where the state (through physicians) paternalistically or in the interests of public health coerces patients in high-risk institutional care settings to receive COVID-19 vaccinations (Schaefer et al. 2022), or where the coercive action seems to exemplify the proper relationship between agents (for instance where a parent makes their child stay out of trouble).

### 2 Analysing Three Case Studies from the COVID-19 Pandemic

As we have highlighted already, during the COVID-19 pandemic, the concepts of solidarity and coercion came up frequently in political and public discussion. We have selected the following case studies to represent both 1) uses of solidarity and coercion rhetoric, and 2) identify solidaristic and coercive practices, in a descriptive sense. The cases below highlight how solidaristic or coercive actions can come apart from the normative judgements that are often assumed in association with them.

In the following case descriptions and analyses, we hold that the cases constituted an accurate use of rhetoric, inasmuch as the signifier (‘coercion’ or ‘solidarity’ labels) co-occurred with the signified (actual coercive or solidaristic action). We remain neutral as to whether the policy was right overall, but argue that the policy was at least not rendered wrong *due to its coerciveness*, and not rendered right *due to its solidarity-promotion*. Whether right or wrong, the normative valence of the measure comes from the way it promotes or undermines justice, whether it effectively protects health through reducing person-to-person contact and thereby transmission, etc. These concepts (and others) that have real moral valence are the appropriate terms to use in public health ethics analysis when evaluating the permissibility of the action, rather than solidarity and coercion.^3^

#### 2.1 Case study 1 – Post-COVID-19 school reopenings in the USA

By March 24^th^, 2020, all states in the USA had implemented school closures, many of which lasted for several months of that year (Education Week 2020). Then, in 2021, many schools that had been operating online or in hybrid format were required by the Department of Education to reopen their doors to students (Christensen 2020). One might expect a warm welcome for such a measure, which could be framed as getting rid of a restriction. Yet, whilst this might be viewed from one perspective as the relaxation of a coercive public health measure (that is, ending compulsory school closures and online-only learning), from another perspective, the *order* to reopen (accompanied by requirements for testing, mask-wearing, and contact-tracing, and penalties for teachers’ non-compliance) is in itself a coercive action. We hold that the label of ‘coercion’ attached to such an action is a legitimate use of the signifier where the signified is present. The order to reopen was given by the Department for Education but was based on guidance from the White House and the US CDC, citing the benefits of in-person education, reduced risk of severe illness in children, and increasing vaccination rates. Teachers who failed to comply were at risk of losing their jobs. In some cities, this led to significant protest action by teachers, including in New York, where schools were set to open earlier than other states (Kopp et al. 2021). The protests and union action resulted in delayed reopening (4 New York 2020), but there were still concerns among teachers about their health, the risk of their spreading COVID-19 picked up via school children, and the lack of plausible options for them to refuse to comply with the order.

In applying the descriptive account of coercion that we have defended in the section above to this case, we hold that the case constitutes coercive practice because there was threat of job loss for those who failed to comply with the action, and the state used the power differential between state and citizens (teachers) to back this threat.

Some media articles discussing these efforts to reopen schools use the language of teachers’ rights being violated (Walsh 2020), or their freedoms curtailed. Legal investigations also discuss whether rights violations may have occurred, making comparisons to employment discrimination due to disability status (Foreiter 2021). These media do not simply mis-label the occurrence of coercion where it is not happening: the implicit threat of one’s job being lost backs up an interpretation of school reopenings being coercive according to our definition of the concept: the threat effectively narrows the options available to teachers in such a way as to shape their action to the coercer’s will. It is a particularly interesting case because it balances the mental health, educational, and overall wellbeing gains for children from in-person school education against the personal health concerns and concerns for spreading disease to others of adult teachers. For teachers, the reason this trade-off was wrong was presented with an emphasis on the *threat*, and their being effectively forced to comply. The coercive nature was the focus for moral condemnation of the action. However, the focus for saying this trade-off was wrong could have been more appropriately on the justice and utility questions raised by the trade-off between children’s and adults’ needs, and between education and mental health *vs* physical health. We hold that these latter two morally relevant factors are appropriate for judging the rightness or wrongness of school reopenings—not whether or not they were coercive.

For our purposes here, we do not need to make an overall judgement of whether the measure was morally permissible or not; rather, what is notable is the emphasis on coercion rather than these other, properly normative considerations. If there were no questions raised about whether it is fair to trade off children’s *vs* adults’ needs, or whether it is more valuable/higher utility overall to promote health-related wellbeing or education-related wellbeing, then would there have been an issue raised with regard to the coercive nature of this measure? It seems that here, coercion is acting as a proxy or even a mask for the actual intrinsically reason-giving considerations of justice and utility.

Our claim might be demonstrated by comparison with the original measure: school closures were undoubtedly coercive of families and teachers alike, though, at least for families, not through the use of threat, but through simple restriction of options: with no teachers available, parents simply could not send their children to school to learn, they did not have that option. This mirrors the differences in means of liberty restriction discussed in relation to other pandemic measures (Giubilini 2023). By contrast with the significant union action and media coverage in response to schools reopening, there was little written or protested about the school closures–perhaps because the trade-offs seemed, in that case, more clearly justified as just and as effective for protecting population health (despite both this effectiveness and the fairness of sacrificing children’s education for population health having been questioned later in the pandemic). Initially, at least, the sacrifice of in-person education seemed a small price to pay for health protection, including of the teachers, people they might be shielding, and parents. We were not initially as aware, either, of the significant educational difficulties, lack of social development, and mental health deterioration that would arise from children’s lack of in-person education during those years, for many age groups (Munro et al. 2023). As a result of seeing the trade-off as more clearly resolvable in the direction of school closures, their coerciveness was not highlighted as an issue. It did not, at that point, appear morally valent. So why was it used as a moral judgement for the later school reopenings? The alignment of the neutral response to coercion with a perceived easier value trade-off, and a negative response to coercion in response to a perceived harder value trade-off may indicate that coercion is being used as a proxy or label for actions perceived to be morally impermissible when, in fact, it is not the appropriate or real issue in question, ethically. Coercion is, in cases like this, appropriately seen as a mechanism by which an action is taken, a description, rather than a normative judgement in itself.

#### 2.2 Case Study 2 – UK COVID-19 self-isolation order and lockdown

On March 12^th^, 2020, the UK government announced a self-isolation order, requiring anyone with symptoms of COVID-19 to isolate (UKHSA 2020). In his announcement of the new policy, then-Prime Minister, Boris Johnson, said those with symptoms “should stay at home for at least 7 days to protect others and help slow the spread of the disease.” (Prime Minister’s Office 2020). The UK public were told that in following the order they would “protect the NHS and save lives” (Cabinet Office 2020). According to the Prime Minister, at that point, “The most important task will be to protect our elderly and most vulnerable people” (Prime Minister’s Office 2020).

As the full extent of the UK outbreak became clear faster than expected, it was only a week and a half before this new policy was followed by a population-wide stay-at-home and social distancing order, i.e., a lockdown (Cabinet Office 2020), which rendered the previous voluntary self-isolation order irrelevant. Police were then given powers to issue fines and disperse gatherings, in accordance with law underpinning the new policies (The Health Protection (Coronavirus) Regulations 2020; Coronavirus Act 2020). Versions of this first self-isolation order remained in operation between UK lockdowns through 2020 and into the second half of 2021 (NPCC 2021). The role of police in enforcement included assisting with cases where individuals had left managed quarantine facilities and taking infectious individuals for screening under exceptional circumstances (NPCC 2021). It seems that there were two reasons given for people in the UK to comply with the self-isolation order and subsequent lockdown: first, the threat of penalty associated with coercive action (police enforcement and fines); second, solidaristic values (protecting the vulnerable and the health system). Despite Queen Elizabeth II giving a royal message a short time after PM Johnson’s appeal centring on unity and coming together to protect the vulnerable (BBC 2020), Brexiteer ministers were at the same time asking Johnson to “also make clear we will not go down the road of coercive lockdowns ever again” (Walker et al. 2022, n.p.).

This case contains both coercive action and solidaristic action (or at least call for solidaristic action) according to our account. Regarding its coerciveness, there is both signifier (the label of coercion, attributed by Brexiteer ministers in the quote above), and signified (the existence or content of coercion, present in the use of threats, power differential exploitation, and the restriction of options experienced by people during lockdowns) present in this case. The UK COVID-19 self-isolation order was coercive according to the definition we have given based on Anderson (2010), and according to other accounts such as Nozick’s threat account (1969). It exploited power structures between the UK government and UK residents to change their behaviour, and it employed both threat (of fines) and force (police powers to return people to their homes). The same goes for the fit of solidarity as both signifier and signified in this case. People did indeed identify themselves as a group with either a shared characteristic (UK residents vulnerability to COVID-19 infection and its consequences) or a shared interest (preventing health, economic, and other losses from COVID-19); they did see a need to promote their shared interest in stopping the spread of COVID-19, and in this case, a need to protect the vulnerable within the identified group of UK residents, a hallmark of solidaristic action, as well as a descriptor of it as used by the Queen. What is interesting in this case is the intersection of coercion and solidarity as each contributing *pro tanto* rightness or wrongness. For instance, the UK government’s ‘we’re all in this together’ during COVID-19 may have been perceived by many as an appeal to solidarity that made subsequent restrictive public health actions morally better. The need for this moral betterment to begin with might have been perceived to arise from the use of a coercive action that, through 15omewhatn, was deemed at least 15omewhatt wrong.

First, we will assess the action through the lens of what we consider to be morally valent concepts of effectiveness at health promotion, and justice. Effectiveness at health promotion (or, we might say, health benefit) is one morally relevant external factor that might render a coercive measure preferable to alternatives. If we think that effectiveness is morally important in justifying a public health measure, and holds a normative valence that coercion does not, then it may be relevant to consider evidence in favour of effectiveness. If it is indeed the case that mandatory isolation measures can be effective at reducing transmission of COVID-19 whilst being less harmful than lockdowns (Iezadi et al. 2021), and particularly so when paired with digital contact-tracing (Haug et al. 2020; Leung et al. 2022), then this evidence of effectiveness might speak in favour of mandatory isolation measures. We do not wish to use this evidence to judge in favour of the measure overall, but to note that effectiveness at promoting health, or health benefit, is one consideration. Insofar as health is intrinsically valuable, health benefit can be ascribed a moral valence which coercion cannot.

Justice is another important normative concept. Coercive measures can promote justice where they alter an unfair distribution of health outcomes for the better, or where they impose burdens on the population to protect health in a fairer way than the burdens of health protection would befall the population without coercion. We do not have the space or access to the evidence needed to fully evaluate whether the coercive self-isolation measure promoted justice, effectively protected health, and was preferable in these ways to alternatives. It is not the point of our paper to do so; rather, we aim here to highlight the difference in normative status between the terms ‘coercion’ and ‘solidarity’ on the one hand, and terms like ‘justice’ and ‘effectiveness (of health promotion)’ on the other. The evidence might support the application of these latter terms as part of ethical arguments in favour or against the measures, as the case may be. But it is these terms, with their use backed by evidence, not the former, descriptive terms, that ought to form part of an ethical analysis. It doesn’t make sense, intuitively, to talk about justice without aiming to promote it. By the same token, although effectiveness *tout court* is not a morally valent term (I might very effectively torture someone, or I might very effectively protect them from harm), effectiveness at health promotion appears to be. After all, health is often understood–either intrinsically or instrumentally–as a good in-and-of-itself. The term coercion is markedly different; for those of us who wouldn’t call ourselves libertarians, the use of threat or power differentials, or changing the structure of choices in order to limit freedom is not intrinsically a bad thing. Its moral valence depends on the goal of the action.

The remaining question is whether the solidaristic justification put forth for self-isolation order in the UK during the COVID-19 pandemic was morally relevant, acting as a *pro tanto* reason in favour of the measure. We do not see solidarity as playing this role. As Case 3 below particularly highlights, but Case 2 also shows, solidarity can create in-groups and out-groups that result in exclusion and mistreatment of marginalised populations at the same time as it aids in the protection of other vulnerable populations. Whilst we may intuitively think of solidarity as a good thing, it was clear during the pandemic that in fact not every action that brings together a group with a common interest (like the British public) in the sharing of a common burden for the protection of a vulnerable subset of the population is good. Self-isolation orders often negatively affected other vulnerable groups, including multigenerational households in areas with larger ethnic minorities in the UK. These tend to be poorer and more crowded households (Mahmood et al. 2021), meaning that those within such households may be exposed to greater harm from having to have infected individuals isolate in a shared household with older relatives. This was ‘solidaristic’ insofar as it constituted an effort to protect the elderly and immunocompromised from severe infection, but the most vulnerable among the elderly—those living in multigenerational households in socially deprived areas--were not considered. Again, we do not wish to make any overall moral judgement of the measure as a whole here, but merely to highlight that solidarity is not necessarily a good thing, a reason in favour of pursuing a public health measure. Any solidaristic measure, like a coercive measure, ought to be evaluated on the basis of truly morally relevant factors rather than these morally ambiguous terms of solidarity and coercion, which ought to be used, instead, descriptively.

### 2.3 Case study 3 - EU Vaccine Procurement during COVID-19

Solidarity was frequently invoked in efforts to promote equitable access to COVID-19 vaccine. The WHO framed solidarity as a moral imperative to overcome “vaccine nationalism”, ensure equitable access to medical countermeasures and underscore a global, collective interest in controlling the pandemic (Eccleston-Turner and Upton 2021; Yamey et al. 2020). Solidarity also underpinned the creation of the COVAX facility (Yamey et al. 2020). This vision of solidarity was an inclusive one intertwined with justice.

Simultaneously, the European Union (EU), invoked solidarity as an ethical basis for ensuring vaccine access for a narrower, more inward-looking group: themselves (European Commission 2020a; European Commission 2020b). The EU framed its collective procurement of vaccines as an expression of solidarity (de Mata, 2020; European Commission, 2020a).

In applying to this case the descriptive account of solidarity that we have defended in the section above, we hold that the case constitutes solidaristic practice because there was an identified group with a shared characteristic—residents of EU member states who were vulnerable to the effects of COVID-19 and who could together afford vaccinations—and the enactment of an action to promote their shared interest—the buying up of vaccines from suppliers. This signifier was accurate, solidarity was present in the action—only, solidarity pertained exclusively with EU member states, without regard for the consequences this exclusion would have for others. The EU actively advised states against joining global initiatives like COVAX and instead established its own joint procurement mechanisms and placed the limitations on moving COVID-19 goods out of the EU (Eccleston-Turner and Upton 2021). This regional solidarity was justified as a means of preventing competition between wealthier and poorer EU countries and while ensuring timely access to affordable vaccines across the EU (Eccleston-Turner and Upton 2021). While this solidarity benefitted EU member states, it had negative consequences for low- and middle-income countries. The monopolisation of vaccine doses by wealthier countries created scarcity and little to no access to vaccines in low- and middle-income countries. This was exacerbated when the EU, having secured a substantial share of the global vaccine supply, implemented export controls to prioritise the immunisation of its own population (Eccleston-Turner and Upton 2021). Part of the reason the EU was able to secure these arrangements was due to its economic power, something LMICs did not share and could not leverage to get access to vaccines.

One of the clearest illustrations of the inequity that characterised this regional self-protection was when South African production facilities contracted to “fill and finish” Johnson & Johnson vaccine doses were required to export those doses to the EU when few (if any) South Africans had received even a single dose (Forman et al. 2022). This exemplified the inequities that get exacerbated by leveraging solidarity to protect privileged groups.

This example of solidarity illustrates that, absent justice, solidarity is not morally valent. It is where solidaristic action promotes justice and other values that we think it good. While solidarity was frequently (and applicably, without mere rhetoric) invoked in support of vaccine equity, and as inherently normatively positive, this case illustrates how such solidaristic action, when it does not promote justice, may be harmful or negative. It also illustrates the need to consider solidarity’s operation beyond being a means of the powerless or disempowered collectively organising to obtain power (Zheng 2023). That is to say, we must consider solidarity when used by the already powerful, and without regard to justice, not solely as a means to empower the disempowered. In this regard, insofar as valence can be layered on top of a description of solidarity, this valence is integrally linked to power (of those acting in solidarity and those not), exclusion and justice. The creation of in/out groups is part of the history of solidaristic action – the working class rising against the bourgeoisie – but, without mind to asymmetries in power and resources, it can quickly become a means to marginalise the already vulnerable and poor. Solidarity by the powerful against those less powerful may well be appropriately layered with negative moral valence. In this case, solidarity created a ‘we’ within the EU, while simultaneously reinforcing structural injustice at the global level. Rather than resulting in a more equitable, safer and healthier world in a way that unified countries, the EU’s version of solidarity served to deepen divisions.

This case study also underscores the political nature of solidaristic action. Prainsack and Buyx have noted that solidarity is not merely a moral sentiment but entails decisions about who is included, who is excluded, and what obligations follow (2017). In exercising solidarity, the EU created a ring-fenced community entitled to solidarity and, correspondingly, gave legitimacy to prioritising their interests over others’, even when that decision produced harm elsewhere, and for a more disadvantaged group. Here, solidarity functioned as a justificatory frame for exclusion along lines of power. Solidarity was not a challenge to global vaccine inequity, but a means of sustaining it (Forman et al. 2022). This reveals how solidarity can reinforce power dynamics and legitimise injustice. One cannot assume that solidarity is good, or even just beneficial for public health when it is operationalised within a broader geo-political context.

## Conclusion

The way we usually talk about the concepts, solidarity is generally thought a good thing, and coercion is generally thought a bad thing. This association between each concept and a moral valence highlights two possibilities (namely, solidarity as ‘good’ and coercion as ‘bad’) at the cost of rendering less visible the other two possibilities (namely, ad solidarity as ‘bad’ and coercion as ‘good’). The goodness of solidarity is commonly attributed and is demonstrated in Cases 2 and 3 above in the discussion of, first, lockdowns against infectious disease to protect the vulnerable as solidaristic, and, additionally, of vaccine procurement and protectionism within a bloc of nations as solidaristic. Both were presented and perceive positively insofar as they were solidaristic. In parallel, the badness of coercion is commonly attributed, as demonstrated in Cases 1 and 2 above, in the discussion of both school

reopenings as coercing teachers, and of lockdowns that restrict UK citizens’ movements and threaten penalties as coercive. Both were presented and perceived negatively insofar as they were coercive. The possibilities of ‘bad’ solidaristic action or ‘good’ coercive action are a rarity in discussion. Yet, as our analysis of the case studies above demonstrates, we can de-couple solidarity from moral goodness, and coercion from moral badness (Table 1).

As Table 1 demonstrates, both solidaristic action and coercive action could be good, or could be bad, yet whilst good solidarity and bad coercion are often recognised, the possibilities of good coercion and bad solidarity are under-recognised. If the attribution of these moral labels of good or bad depends on the context and application, then they are better off considered descriptive concepts. Whilst it may be relatively uncommon that coercive actions are good and solidaristic actions bad, it shows how the two terms are better used descriptively, and are frequent *proxies or correlates* for moral considerations such as justice and effective provision of primary goods, rather than being moral considerations themselves. To conclude, consider the example that originally inspired this work, but which we didn’t analyse above. When South Africa shared its epidemiological data on COVID-19 cases with the rest of the world, the solidaristic nature of this action was seen to, but in fact did not render it morally good (Silva and Smith 2022). In the context of many high-income countries breaking promises to reciprocate data-sharing efforts through access to vaccines and other benefits, and when trust is subsequently undermined through vaccine hoarding, solidaristic action may not be good or justified, insofar as it exacerbates existing unfair health inequalities. It is essential that bioethics work like that by Silva and Smith continues to be done. Bioethicists must sensitise themselves to the possibility of good coercion and bad solidarity, and to avoid falling into the trap of attributing normativity to concepts that ought to be used descriptively. This sensitivity can add nuance not only to the bioethics literature that critiques public health measures, but also to the ways that ethicists can inform policy decision-making and the use of rhetoric. Over time, we hope that further academic reflection on this issue will reduce the use of descriptive terms to normatively justify actions in political rhetoric as well as academic debate. Certainly, more accurate use of these terms is important for trustworthy, transparent and ethically appropriate policy responses to future health emergencies.

## Figures and Tables

**Table 1:** 

Moral valence/concept	Solidaristic action	Coercive action
**Good**	ATTRIBUTED	UNDER-RECOGNISED
**Bad**	UNDER-RECOGNISED	ATTRIBUTED

## Footnotes

1 Exploitation also involves leveraging power differentials to curb action, and has a complex interrelationship with coercion; investigating this related term is outside our scope here.

2 Wertheimer holds it does, though note that he is concerned primarily with the moral implications for the coercee, i.e. whether they should be held morally responsible for performing coerced bad actions (Wertheimer 1987).

3 We also dispute the positions of libertarians who think the infringement of freedom of movement and association wrong under any circumstances (or at least under these circumstances, where the goal was to protect the health of others and slow the spread of disease); and we dispute the primacy of a presumption in favour of liberty held by many including the Nuffield Council on Bioethics (NCOB), which released a rapid policy briefing on March 17th (2020, 1) holding that “Coercion and intrusion into people’s lives should be the minimum possible consistent with achieving the aim sought.”
